# P-2037. Epidemiology, Clinical Features and Outcomes of COVID 19 Patients Post Evusheld Therapy Among Immunosuppressed Patients

**DOI:** 10.1093/ofid/ofae631.2193

**Published:** 2025-01-29

**Authors:** Pramodini Kale-Pradhan, Sarah Channey, Mamta Sharma, Daniel Lebovic, Christopher Giuliano, Leonard B Johnson, Ashish Bhargava

**Affiliations:** Wayne State University Eugene Applebaum College of Pharmacy and Health Sciences, Detroit, Michigan; Wayne State Unvieristy Eugene Applebaum College of Pharmacy and Health Sciences, Detroit, Michigan; Henry Ford St John Hospital, Grosse Pointe Woods, MI. 48236, Michigan; Ascension St John Hospital, Detroit, Michigan; Wayne State University Eugene Applebaum College of Pharmacy and Health Sciences, Detroit, Michigan; Ascension St. John Hospital, Detroit, Michigan; Ascension St. John Hospital; Wayne State University School of Medicine, Grosse Pointe Woods, Michigan

## Abstract

**Background:**

Evusheld® is a combination of two monoclonal antibodies, Tixagevimab and Cilgavimab. It was developed for the pre-exposure prophylaxis (PrEP) and treatment of COVID-19 illness for those who are immunocompromised. There is paucity of long-term real-world data on the use of Evusheld among the immunosuppressed patients against the SARS-CoV-2 Omicron variants.

**Methods:**

A retrospective cohort chart review of patients 18 years and older who received the two dose Evusheld regimen from December 1, 2021 to January 31, 2023 at a community teaching institution was performed to evaluate the effectiveness of Evusheld as PrEP of COVID-19 patients. Evusheld as PrEP was indicated for patients with active solid tumor and hematologic malignancies or those receiving immunosuppressive treatment including CART therapy, biologic agents and high-dose corticosteroids (i.e., ≥ 20 mg prednisone or equivalent per day when administered for ≥ 2 weeks). The following data was collected: patient demographic, development of COVID19 after receiving Evusheld®, intensive care unit (ICU) length of stay (LOS), hospital LOS, ventilation requirement, duration of ventilation, and mortality among these patients within six months of receiving Evusheld therapy.

**Results:**

Of the 663 patients screened, 459 were excluded primarily for duplicate patients. The mean age of the 204 included patients was 68.3 years and 97 (47.5%) were female. Eighteen (8.8%) patients had a positive COVID-19 test within 180 days of receiving Evusheld. None of the COVID positive patients were admitted to the hospital or required mechanical ventilation. All COVID positive patients survived.

**Conclusion:**

Our study shows that Evusheld was effective in preventing COVID-19 among the immunocompromised patients during the six-month follow-up period. This study also showed that patients who developed COVID-19 after Evusheld did not develop severe disease or require hospitalization.

**Disclosures:**

All Authors: No reported disclosures

